# Hereditary and Acquired Characteristics as Social Questions

**Published:** 1901-11

**Authors:** R. R. Kime

**Affiliations:** Atlanta, Ga., Ex-President Tri-State Medical Society, Ala., Ga. and Tenn.; Ex-President Atlanta Society of Medicine; Member American Medical, Southern Surgical and Gynecological, Georgia State Medical Societies; Chairman Sociological Com. Tri-State and State Societies


### HEREDITARY AND ACQUIRED CHARACTERISTICS AS SOCIAL QUESTIONS.

BY R. R. KIME, M.D., ATLANTA, GA.,

Ex-President Tri-State Medical Society, Ala., Ga. and Tenn.; Ex-President Atlanta Society of Medicine; Member American Medical, Southern Surgical and Gynecological, Georgia State Medical Societies; Chairman Sociological Com. Tri-State and State Societies.

In the elevation or degradation of the human race we have but two primal factors, that which is transmitted or that which is acquired.

The exact relation of the acquired to the transmitted and the possibility of the acquired to a certain extent being merged into the transmittal are as yet unsettled questions.

It is acknowledged by all that heredity is a potent factor in the building up or tearing down, not only of the animal kingdom but the vegetable kingdom as well.

It is a vital principle, far reaching in its possibilities, and fully confirms the statement that "the sins of the father are visited to the third and fourth generation."

Heredity manifests itself through the anatomical, physiological, and psychological aspects of mankind, and in either may be normal or pathological. (American Text Book Physiology, Vol. II II, p. 494.)

That hereditary characteristics are transmitted, and within certain limits may elevate or degrade the race, all admit; but, as to acquired characteristics becoming hereditary, there is difference of opinion.

The relation of hereditary and acquired characteristics to each other, and their influence upon the human race, involves social questions of far reaching importance and well worthy of study and serious consideration.

"Heredity includes whatever is transmitted, either as actual or as potential characteristics, by parents to offspring."

The transmission of hereditary characteristics is said to be scientifically demonstrated by Weismann.

Such characteristics being transmitted through the germplasm (idioplasm of Nageli), and that the nucleus alone of the germ cell is transmitted more strictly that the chromatic substance of the nucleus is the sole actual germinal substance.

In order to carry out the theory that hereditary characteristics only are transmitted, Weismann denies the theory of pangenesis, *i. e.*, the production of germ-plasm in the body as maintained by Darwin, Spencer, and others. Weismann claims that the germ-plasm is not produced from the body tissues, but has its sole origin from the germ-plasm of the parent of the individual. Through the parent from the grandparent, and so on, may be traced backward through families and tribes and races to its origin in simple unicellar organism. (Amer. Text Book Phys., p. 499.)

It is further stated that His and Weismann hold that the germ-plasm possesses a complicated architecture. That germ-plasm is stable in composition, transmitted from one generation to another, in fact immortal and essentially distinct from somato-plasm (body-plasm), which develops anew in each generation from the germ-plasm, being variable, and dies when the subject dies.

Thus in brief we have the theory of hereditary transmission demonstrated to the exclusion of acquired characteristics.

While with our present knowledge it may be difficult to demonstrate scientifically the transmission of acquired characteristics, there is certainly sufficient evidence at least to justify the assumption that such characteristics are in some instances transmitted.

The human race and each genus of the animal or vegetable kingdom is subject to improvement or degradation within certain limits. To accomplish either result some would have us believe only that which existed in the first primitive being from the first origin of life can be transmitted and not the acquired. That the improvement or deterioration of each genus is wrought by variations in the original primitive stock of heredity.

This would be a fatal blow to evolution, because then each species would necessarily require a special act of creation in order that the first germ cell of each genus might contain all the hereditary possibilities of that genus. Otherwise every germ cell would have locked within its narrow confines all the elements necessary to the development of a mouse, elephant, reptile, whale, man or fowl, *ad infinitum.*

Such a germ cell would require infinite knowledge to prevent wrong development in the different genus or species, else we must admit that the present germ cell of man is entirely different in its make-up of possibilities and architecture from that of the worm, the dog, the fowl, or fish. If they differ, new characters have been acquired and the Weismann theory of the non-transmission of acquired characteristics falls.

Again, in the lower order of animal life we have the bisexual, in the higher order the unisexual development. Whence the change or variation except by acquirement?

If the germ cell of man differs from that of the lower order of animals and the germ cell of the unisexual from the bisexual, then it is self-evident that acquired variations at least have been developed and transmitted.

If acquired characteristics are not transmitted, then there is no such thing as transmission of hereditary diseases or hereditary predisposition to certain diseases.

It is a well established fact that some families have a predisposition to certain diseases while others have not, which could only exist by acquirement and transmission from one generation to another.

How can such serious results be transmitted to the child begotten in a drunken debauch of parents except by acquirement? (Amer. Text Book Phys. p. 503).

It is stated in the Weismann theory that the germ-plasm is not increased nor diminished by passing through successive generations and can be traced back without change to its origin in unicellular organisms.

From this statement we are expected to believe that the germ-plasm of all the different races of mankind are one and the same, that the germ-plasm of all the lower order of animals, from the monkey down to the amœba, are one and the same with that of man. That in the chromatin of nucleus of the germ cell of the germ-plasm of every man, of every animal on the earth, of every living thing in the sea and all the fowls of the air contain locked up within such a tiny cell all the possibilities and the architecture necessary to develop any one or all of these living creatures.

That the same germ cell which exists in you or me is the same as that which existed in Adam or in our colored brother, though his skin is black.

That this same germ cell or germ-plasm exists in the horse we drive or in the pig, chicken and fish we eat, hence we are cannibals if the Weismann theory be true. As for myself, I prefer to accept the transmission of acquired characteristics as possible and wait for its scientific demonstration, or accept tentatively the theory of pangenesis as advocated by Darwin.

It is self-evident at least that we exist today in different surroundings and acquirements from that of the savage or primitive man. Our present state of civilization, enlightenment, ideas of social relation, and knowledge of scientific questions, are an evolution at least from a lower order of things.

Such evolution is not, never has been, and never will be within the province or possibilities of any germ save that of man, because none other is endowed with the same powers and possibilities.

Whence these powers and possibilities came has been the subject of metaphysical speculation for ages past and will continue to be for ages to come.

Man, the masterpiece of creation or the climax of evolution, is subject to elevation or degradation by heredity, environment and acquirement.

As a social being we must consider all three of these factors in attaining the noblest and best for mankind.

The development of dwarfs and giants, as well as various abnormalties of muscular and oseous systems are frequently due to transmitted tendencies.

We are of the opinion that acquired characteristics to a certain extent under certain conditions are transmissible. In many instances we have the transmission of disease or predisposition to disease. In gout 90 per cent. (Gairdner & Garod-Zeigler Pathology). Consumption 38 per cent. (J. A, Robinson-Jour. A. M. A., p. 505). Insanity 25 to 85 per cent, (Zeigler Pathology, p. 94). Obesity, epilepsy, and insanity are considered hereditary, as well as various psycophatic and neuropathic conditions. Butler names over 40 diseases and conditions hereditary. P. 14 Diag. Inter. Med.

The laws governing inheritance are for the most part unknown. Darwin Origin Species, p. 15.

If strange and rare deviations of structure are really inherited, less strange and commoner deviations may be freely admitted to be inherited. Unless favorable variations be inherited by some at least of the offspring nothing can be effected by natural selection (Darwin Origin Spe. Vol. 2. p. 246). Man can and does select the variations given him by nature and thus accumulates them in any desired manner. He thus adapts animals and plants for his own benefit and pleasure. (Darwin p. 277.).

Talbot has noted congenital absence of the foreskin in 3½ per cent. of the Jews.

"There is no doubt whatever that the criminal parent tends to produce a criminal child," says Havelock Ellis, The Criminal, p. 99.

When we see in various tribes and families of mankind originating in one common stock such diverse family or tribal characteristics, that did not manifest itself in the original, we are forced to believe they have been acquired.

When we see in the animal kingdom such different characteristics as are manifest in the pacer, trotter, running, light harness and draught horse, in the pug, water spaniel, greyhound, shepherd, setter and bulldog, such distinct characteristics in the various species of pigeons, with the endless variety of new productions in the vegetable kingdom, considering the extent to which each may be developed or degraded, and the various diseases that may be ingrafted and predispositions to such be transmitted, we are forced to believe to some extent at least in the transmission of acquired characteristics. Not all acquired characteristics are transmitted neither are all hereditary characteristics transmitted, in any one given case or species.

As a working basis, we suggest the following as plausible, practical and consistent. *Acquired individual, permanent characteris-*

*tics, produced through natural or perverted functions of the body become hereditary in character. The earlier in life such characteristics are acquired, the greater the tendency to transmission, and when carried through successive generations tend to become permanent hereditary characteristics.*

Accepting the above proposition, we can readily understand how it is possible to permanently elevate or degrade any genus or species of the animal or vegetable kingdom by systematic, scientific methods. Unless acquired characteristics are transmitted to some extent we have not the same incentive to work for the improvement of the race.

As an illustration of our point let us state a hypothetical case.

Take two boys born of healthy normal parents, without disease or taint, morally or physically, and raise one with high moral surroundings, the best of physical development, influenced by all that is good and noble, through only three successive generations, and compare his offspring with the other boy, who from his youth has been placed under bad moral surroundings, taught by precept and example, in the use of alcoholic beverages to excess through only three successive generations, and note the contrast. One will be a noble specimen of true manhood, the other a drunken sot, else a mental or physical degenerate, diseased and degraded. Is it reasonable to suppose all this change comes from heredity alone and none from acquirement?

We cannot help but believe that observation and experience confirm the theory of transmission of acquired characteristics whether they be good or bad.

We also believe that the acquired characteristics tend to become hereditary in an increasing ratio—that is, the acquired of one generation will to some extent become the hereditary of the second generation, while the acquired of the second will to some extent add to the hereditary of the third, thus tending to develop the good or bad in an increasing power.

Thus a nation builds up or goes down with increased momentum according as these principles are utilized by its subjects. The question that concerns us most, what are we as a nation or profession doing to utilize these powerful factors for the uplifting and upbuilding of humanity?

What is our destiny as a people and as a nation? Can any one tell?

It depends upon what use we make of the means at our command to regenerate, purify and uplift ourselves.

A blind man can read our destiny if we allow the vices and evils of this day to go on unchecked and uncontrolled.

Do I hear some one say the world is growing better every day? If so, let those speak who have made a study of this question.

"The level of criminality is rising, and has been rising during the whole of the present century, throughout the civilized world. (Ellis—The Criminal Pa. 259.) In France, in Germany, in Italy, in Belgium, in Spain, in the United States, the tide of criminality is becoming higher steadily and rapidly. In France it has risen several hundred per cent., in Spain the number of persons sent to perpetual imprisonment nearly doubled between 1870 and 1883, in the United States the criminal population has increased since the war relatively to the population one third.

Brower—"We are surprised at the rapid increase of crime, rapid much beyond the increase of population. In 1850 the ratio of prisoners to the population was 1 in 3,442, in 1890 was 1 in 757. In 1890 the number of criminals in the United States was about one-quarter of a million.

In Pennsylvania from 1880 to 1890 population increased 22.5 per cent., criminals 34.7 per cent. New York city in ten years population increased 33⅓ per cent., crime more than 50 per cent. In Chicago the number of arrests in 1884 was 39,434, in 1893 was 96,976. (A. M. A. Jour. June 1899.)

When we consider the way in which the average American lives, his development, the amount of alcoholics, opiates, cocaine, and coal tar derivatives used, the habit of indiscriminate dosing with patent medicines, the free license given criminal degenerates and the diseased to marry and propagate, with the free license of the press to publish the details of assassins, crimes and immoral stories, and even the hot-bed of anarchy allowed to exist in our midst, then it is we fear and tremble for the future of our boasted land of liberty.

It is high time we were looking to the prevention of crime, rather than punishing the criminal, the prevention of vice rather than its control.

The criminal, the inebriate, the degenerate, is often but the result of license.

A license to marry and propagate, a licensed saloon, and vice to develop, and we have the results.

It is said—Lacassagnes'—" That the criminal is a microbe which only flourishes in suitable soils." (Ellis, Criminal, p. 309.)

Also, " Societies have the criminals that they deserve." (Ellis, p. 312.)

McDonald says: " The saloon is the one true home of the criminal. Of 100,000 murders committed in France 2,347 occurred in saloons. Out of 49,423 arrests in New York 30,507 were drunkards." (Criminology, p. 89.)

Brower states: " Fully 50 per cent. of the criminals arrested in Chicago are inebriates; that alcohol is the direct or indirect cause of probably 75 per cent. of all the crime committed."

We quote the following from Ellis. (The Criminal, p. 97):

" Alcoholism in either of the parents is one of the most fruitful causes of crime in the child. There is to-day no doubt whatever that chronic alcoholism, as well as temporary intoxication, at the time of conception, modifies profoundly the brain and nervous system of both parent and offspring."

" Some of the most characteristic cases of instinctive criminality are solely or chiefly due to alcoholism in one or both parents."

" When insanity or alcoholism are combined in the parent, a rich and awful legacy of degeneration is left to the offspring."

" Morel quotes a case—Father alcoholic, mother insane—of five children; 1 commited suicide, 2 became convicts, 1 daughter was mad and another semi-imbecile."

Careful statistics of 4,000 criminals who passed through Elmira, New York, show drunkenness clearly existing in the parents in 48.7 per cent. and probably 11.1 per cent more.

Rossi found out of 71 criminals whose ancestry he was able to trace, in 20 the father was a drunkard, in 11 the mother.

Morro found that on an average 41 per cent. of the criminals he examined had a drunken parent, as against 16 per cent. for normal persons.

"It is not necessary that the alcoholism should be carried so far as to produce great obvious injury to the parent. The action of the poison may be slow and carried on from generation to generation." (Havelock Ellis, p. 98.)

Dr. Dom. Bezzola, specialist in nervous diseases and resident physician in a training institute for imbecile children, states in an address at Vienna: "Judging from the results of the medical investigation thus far, the fact that alcohol injures the human germ of procreation seems well established. I also place myself unconditionally upon the standpoint of my colleagues and believe that the injuries alcohol produces extend beyond the individuality of the drinker to his offspring." His conclusions are based upon the study of a vast number of imbecile children.

Pierce Bailey, M. D., is quoted (Jour. Inebriety, July, 1901,) as saying: "Alcohol is the most important factor in acquired degeneration. Drunkenness of a parent at the time of conception has long been recognized as a fertile cause of imbecility."

"Young man," said Diogenes, "Thy father must have been very drunk when thy mother conceived thee."

N. S. Davis, M. D., states that alcoholics diminish the vitality of parents and promote both the physical and mental degeneration of their children.

Time forbids further enumeration of evidence. Another factor that should not be forgotten in the study of social questions is the age of the parents when children are born.

"Korosi, Director of the Hungarian Statistical Bureau, investigated 24,000 cases and found the children of fathers below 20 are of feeble constitution, that fathers between 25 to 40 produced the strongest children, and that above 40 fathers tend to beget weak children.

"The most healthy children have mothers below 35, between 35 and 40 are 8 per cent. weaker, over 40 are 10 per cent. weaker."

Dr. Morrow has made interesting contributions to the differentiation of various criminal types, and he has brought out very clearly the disastrous tendency to degeneration among the children of parents who have passed the middle age. (Ellis, p. 41.)

So far as heredity contributes to crime, Mr. Boies would meet it by strict marriage laws. He is quoted in Cyclopedia of Social Reform, p. 429, as saying: "The law should strictly prohibit the marriage of females under 20 and males under 25; of males over 45 with females under 40, who have not passed the period of child-bearing, for, outside of these limitations of age, it is generally understood healthy children are exceedingly improbable if not impossible; of vagrants, tramps and paupers, of the insane, idiotic, epileptic, paralytic, syphilitic, intemperate, cancerous and tuberculous; and of the congenitally blind, deaf, defective or deformed."

The more I study this subject the more I am convinced it is our duty to prevent through proper legislation and education the propagation and development of the criminal, the insane, the inebriate and diseased. Such a course is far more reasonable and consistent than to maintain, support and control these unfortunates brought into existence by the sanction and license of the State.

In conclusion, in the name of justice, I ask what moral right has a State to license or sanction the propagation and development of the criminal, the insane, the epileptic, the inebriate or the consumptive?

It cannot be in the name of true liberty, for such offspring are forced into a bondage without their consent, worse than slavery itself.

Genuine true manhood would abhor such liberty. Genuine true womanhood would blush and hide her face in shame at such mockery in the name of liberty. Such is license—license to do evil and violate the Golden Rule of liberty and freedom.

Oh, Liberty, what sins are committed in thy name!

